# Revealing molecular diffusion dynamics in polymer microspheres by optical resonances

**DOI:** 10.1126/sciadv.adf1725

**Published:** 2023-05-10

**Authors:** Jiawei Wang, Jin Li, Shengqi Sun, Haiyun Dong, Lan Wu, Engui Zhao, Feng He, Xing Ma, Yong Sheng Zhao

**Affiliations:** ^1^ School of Electronic and Information Engineering, Harbin Institute of Technology, Shenzhen 518055, China.; ^2^ Key Laboratory of Photochemistry, Institute of Chemistry, Chinese Academy of Sciences, Beijing 100190, China.; ^3^ School of Science, Harbin Institute of Technology, Shenzhen 518055, China.; ^4^ School of Materials Science and Engineering, Harbin Institute of Technology, Shenzhen, 518055 China.

## Abstract

Understanding the diffusion of small molecules in polymer microsystems is of great interest in diverse fundamental and industrial research. Despite the rapidly advancing optical imaging and spectroscopic techniques, entities under investigation are usually limited to flat films or bulky samples. We demonstrate a route to in situ detection of diffusion dynamics in polymer micro-objects by means of optical whispering-gallery mode resonances. Through mode tracking, interactions between solvent molecules and polymer microspheres, including sorption, diffusion, and swelling can be quantitatively analyzed. A turning point of mode response is observed, while the diffusion exceeds the sub-wavelength-thick outermost layer as the radial extent of resonances and starts penetrating the inner core. The estimated solubility in the glassy polymer is consistent with the predicted value using Flory-Huggins theory. Besides, the non-Fickian contribution is analyzed in such a glassy polymer-penetrant system. Our work represents a high-precision and label-free approach to describing characteristics in diffusion dynamics.

## Teaser

Molecular diffusion dynamics in polymer microspheres can be understood by tracking optical whispering-gallery mode resonances.

## INTRODUCTION

Assessing interactions between small molecules in polymers, such as sorption, diffusion, and permeation, as a long-standing research topic, is unquestionably valuable in a plethora of fields such as medicine (*1*), energy storages (*2*), and sensors (*3*). Notably, the diffusion kinetics in a polymer-penetrant system is highly dependent on multiple physical properties, especially the state of the amorphous polymer. It can be classified into three regimes, namely, Fickian (case-I) diffusion in which the rate of penetrant diffusion governs, case-II diffusion in which the polymer relaxation dominates (*4*), and non-Fickian (anomalous) diffusion in which diffusion and relaxation occur with comparable rates (*5*, *6*). In addition to the classical gravimetric technique (*5*), various precision measurement techniques using optical principles have been explored, such as fluorescence imaging (*7*), laser interferometry (*8*), Raman spectroscopy (*9*), dynamic reflection spectroscopy (*10*–*12*), and Fourier transform infrared–attenuated total reflection spectroscopy (*13*). However, most of the characterizations are mainly performed on flat films or periodic yet macroscopic structures. To clarify the diffusion dynamics, especially anomalous diffusion in polymer with tiny mass or scale down to a few micrometers, new methods with improved sensitivity and spatial resolution are highly desirable.

Since the groundbreaking work by Arnold and Vollmer in 2002 (*14*), whispering gallery mode (WGM) optical microcavities have been widely recognized as a highly sensitive detection system with a rapidly extended scope, including physical, chemical, and biological sensing (*15*–*18*). In dielectric microstructures with a circular cross section, such as microspheres (*14*, *19*), microtubes (*20*, *21*), and microrings (*22*), the total internal reflection of light waves along the concave cavity boundary results in efficient optical confinement and high-*Q* WGM resonances (*23*). Primarily, WGM optical microcavity sensors have been exploited for the detection of any solid (bio-)materials in the vicinity of the cavity surface being adsorbed and polarized through interactions with the evanescent field (*24*, *25*). In addition to sensing in an aqueous environment, extensive studies have been performed on gas molecules, sparking applications in vapor sensing (*26*–*30*), humidity sensing (*31*, *32*), and discriminations of volatile organic compounds (VOCs) (*22*, *33*).

In most of the aforementioned cases, the WGM microcavity itself as the transducer is perceived to be “static.” One should revisit this assumption more carefully while the sensor is made by nonsolid WGM cavities (*34*). Under such circumstances, the interaction would no longer be limited to the surface. Hence, the light-analyte interaction does not have to be restricted by the evanescent wave fraction. Notably, the same mechanism applies to WGM cavities made of polymers (*35*–*38*), such as polystyrene (PS) (*39*), poly(methyl methacrylate) (PMMA) (*40*), and poly(*N*-isopropylacrylamide) (PNIPA) (*41*). The polymer-solvent interaction and resulting swelling may occur in both air and aqueous environment over a relatively long time scale and also contribute to the perturbations of optical resonances (*26*, *33*, *41*). In this request, a theoretical model based on perturbative approach was proposed recently to describe the complex process of glassy polymer microspheres immersed in a solvent bath (*42*). Somewhat unexpectedly, the diffusion kinetics of penetrant molecules with varying amounts in polymers have not yet been experimentally addressed with WGM-based optical sensing techniques. Sharp optical resonances in polymer spheres may contribute to fresh insights into understanding molecular diffusion in polymers as a ubiquitous phenomenon, especially those known with complex dynamics in the glassy state or close to the glassy-to-rubbery transition.

Here, we report an experimental observation and quantitative description of diffusion dynamics in glassy polymer microspheres with a diameter of a few micrometers by means of analyzing optical resonances. While previous reports of WGM gas sensors focus on measuring the number of molecules absorbed on the surface, our approach here reveals that optical resonances of polymer microspheres are not only sensitive to the concentration of penetrant molecules in ambient environment but also strongly dependent on the progress of permeation and swelling of the polymer matrix. By real-time tracking of resonant wavelength shifts and mode linewidth changes, different stages of interactions covering the adsorption at the surface, outermost layer-limited diffusion, and further diffusion until saturation can be distinguished. The characterized mode responses indicate a good consistency with perturbative theories and numerical simulation results. Key parameters including the penetrant concentration and solubility in glassy polymer can be extracted by analyzing the mode shifts at different stages. This work represents an approach to understanding the widely existing anomalous diffusion phenomena in polymer microstructures.

## RESULTS

### Principle

**Fig. 1.**
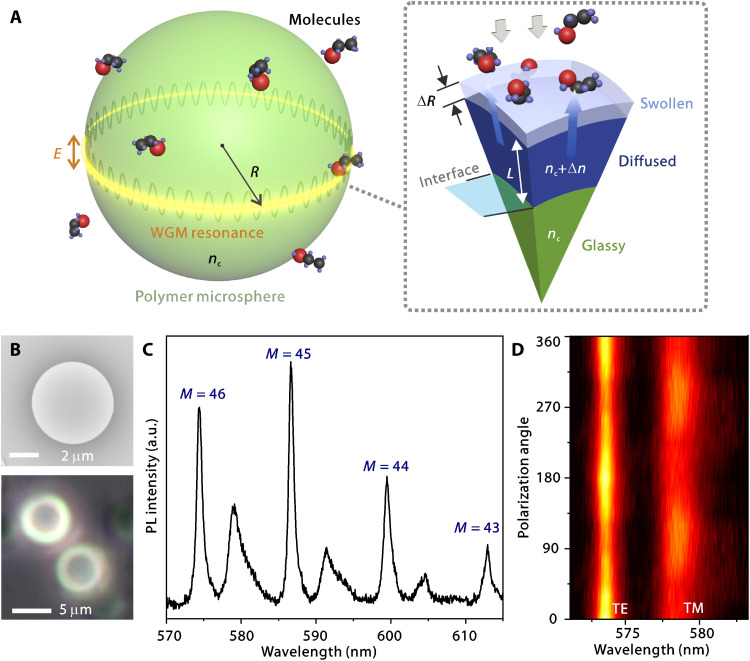
WGMs supported in polymer microspheres are sensitive to perturbations by solvent molecules. (**A**) Left: Schematic of a TE-polarized WGM supported in a polymer microsphere and the interaction with ethanol molecules in ambient environment. Right: Schematic in a cross-sectional view of a segment of the microsphere with the depicted diffusion-caused swollen layer, where the deep blue part stands for the original volume and the light blue part stands for the expanded volume. The undiffused glassy layer at the core region is denoted in green. (**B**) Scanning electron microscopy image and bright-field optical microscope image of PS microspheres. (**C**) Measured resonant mode spectrum containing both TM and TE modes with different azimuthal mode orders. (**D**) Polarization- and wavelength-resolved image showing TE and TM modes. a.u., arbitrary units.

Figure 1 (A and B) illustrates the working principle of analyzing diffusion kinetics in a polymer microsphere by tracking WGM resonances. The resonance frequencies are strongly dependent on the sphere size (i.e., radius *R*) and refractive indices of both the sphere material (*n_c_*) and the environment (*n_e_*). Using an asymptotic approximation, the resonance condition can be expressed as follows (*43*)

$$\frac{\omega_{m}^{\left(l\right)}n_{c}R}{c}=\nu -\xi_{l}{\left(\frac{\nu }{2}\right)}^{1/3}+\frac{n_{c}}{n_{e}}\sum_{k=0}^{k_{\max}}\frac{d_{k}(n_{c},n_{e},\xi_{l})}{\nu^{k/3}{\left(n_{c}^{2}/n_{e}^{2}-1\right)}^{(k+1)/2}}$$ (1)

where ω is the angular frequency of the resonance, *l* is the radial mode order, *m* is the azimuthal mode order, ν = *l* + 1/2, ξ*_l_* denotes the *l*-th zero of the Airy function, and *c* is the speed of light. The coefficient *d*_k_ depends on the polarization state of the light, i.e., the transverse electric (TE) or transverse magnetic (TM) mode [e.g., see *d*_0_ − *d*_8_ for *k* = 0 to 8 in the previous study (*43*)]. As to the fundamental TE mode along the radial direction (*p* = 1) as the simplest case, the mode volume is well confined at the outermost layer of the microsphere (see Fig. 1A).

For a polymer microsphere in the rubbery state exposed to the mixture of air and penetrant molecules, a simple Fickian diffusion model can be applied. While the process temperature *T*_e_ (room temperature here) is notably lower than the glassy-to-rubbery state transition temperature *T*_g_ (~ 94°C; see Materials and Methods), the diffusion dynamics of polymer in the glassy state might be anomalous and also hard to predict because of the slow relaxation (*44*). According to previous studies of non-Fickian diffusion such as alcohol diffused into a glassy polymer (*45*–*47*), generally, a sharp glass-rubber interface can be formed (Fig. 1B), which is attributed to the threshold process in a glassy-to-rubbery state transition (*6*, *48*). The diffusion coefficient *D*_g_ of alcohol molecules into glassy polymers (e.g., ~10^−12^ to 10^−11^ cm^2^/s at room temperature) can be orders of magnitude lower than that in rubbery polymers (*D_r_* ~ 10^−8^ cm^2^/s) (*49*). Hence, the depth of diffusion *L* increases gradually over time, and the microsphere gets swelled with a change of radius Δ*R*.

Assuming a local equilibrium is reached, the volume fraction of diffused penetrant can be expressed as $\eta_{s}=\bar{\nu }U_{0}$, where *U*_0_ is the solubility and *v* is the molar volume (~0.058 dm^3^/mol for ethanol). According to previous reports using gravimetric methods (*45*, *50*, *51*), the interaction between PS and ethanol follows the simple assumption that the volumes of the polymer and the solvent are additive. Therefore, the volume fraction of the polymer can be written as η_c_ = 1 - η_s_. Besides, the diffusion leads to a radial inhomogeneity of the refractive index of the microsphere. As a composite system containing two materials that are randomly interspersed, the refractive index of the diffused layer *n*_d_ can be estimated using the Bruggeman model (see section S1) (*52*). Hence, the change in the refractive index is Δ*n* = *n*_d_ − *n*_c_.

Considering the resonance condition in Eq. 1 that *n*ω*R* ≈ const, the overall effect on the mode resonance wavelength Δλ can be summarized as (*24*)

$$\frac{\text{Δ}\lambda }{\lambda }\approx \frac{\text{Δ}R}{R}+\frac{\text{Δ}n}{n}$$ (2)

Given that the refractive index of the penetrant *n_s_* is usually lower than *n_c_* (e.g., *n_s_* ~ 1.36 for ethanol and *n_c_* ~ 1.59 for PS at 590 nm), Δ*n* is a negative value. Therefore, the two components, Δ*R/R* and Δ*n/n* may contribute with the opposite sign and thus “compete” against each other, leading to distinct responses at different stages of the diffusion process.

### Tracking of diffusion dynamics

WGM resonances of single microspheres were characterized using a confocal microscopic photoluminescence (PL) system (see Materials and Methods and fig. S2 to S4). Two sets of resonant modes can be discerned (see Fig. 1C and fig. S4). Polarization-resolved spectroscopy in Fig. 1D confirms the pair of TE and TM modes at the fundamental radial mode order. The extracted *Q* factor (i.e., the inverse mode linewidth relative to its wavelength, *Q* = λ/δλ) of TE modes is considerably higher than that of TM modes. This is attributed to the potentially higher scattering loss of the TM mode caused by the strong evanescent field interacting with the surface roughness rather than that of the TE mode. To seek the best accuracy of spectral resolution, most of the following studies are conducted on the basis of TE modes.

**Fig. 2.**
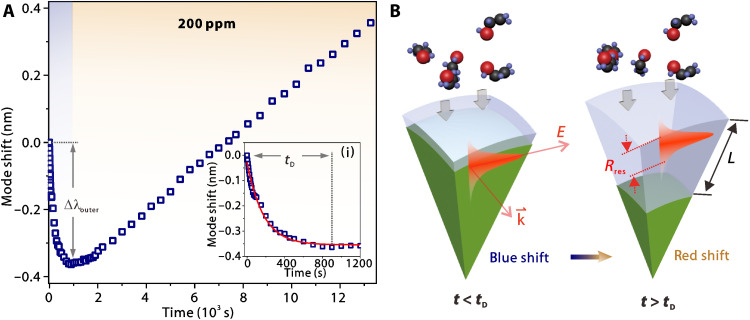
Evolution of resonance modes in a low-load condition. (**A**) Mode wavelength shift as a function of time upon a penetrant concentration of 200 ppm. Inset: Zoomed-in view at *t* = 0 to 1200 s. (**B**) Schematics presenting two stages of diffusion interacting differently with the WGM, respectively.

While the prepared mixture of ethanol and air was injected into a homemade chamber, in situ spectral measurements were carried out (see Materials and Methods). Figure 2A presents the time course of mode wavelength shift at an ethanol concentration of 200 parts per million (ppm; i.e., 8.9 × 10^−3^ mol/m^3^). Initially, the resonances experience a clear spectral blue shift, which contradicts the results in conventional WGM-based gas detection where only red shift was observed (*31*, *33*). When the penetration is limited at the outermost layer strongly overlapping with the WGM field, the mode response is dominated by the refractive index change [see Fig. 2B (left)]. The shift gradually reaches a saturated blue shift value of Δλ_outer_ ~ −0.36 nm at *t*_D_ ~ 900 s.

By considering the mode distribution of WGM resonances together with Eq. 2, the mode responses can be theoretically studied. Apart from the diffusion and swelling, slow relaxation of the polymer may occur as well. A complete analysis considering both effects of diffusion-induced swelling and polymer relaxation is given in section S2. According to previously characterized volume relaxation of PS by dilatometer (*53*) and the prediction of refractive index change using the Lorentz-Lorenz equation, the calculation shows that polymer relaxation makes a very minor contribution in the mode responses and hence can be negligible. Therefore, for simplicity, the following analysis adopts the approximation of neglected relaxation-induced effects. Applying the asymptotic approximation in Eq. 1, the radial extent *R*_res_ can be expressed as

$$\frac{R_{\mathrm{res}}}{R}\approx \frac{{\left(3\pi \right)}^{2/3}}{2}{\left[\frac{q-1/4}{n_{c}k_{0}R_{0}}\right]}^{2/3}$$ (3)

While *L* approaches *R*_res_, the maximum shift can be derived from Eq. 2 as

$$\frac{\text{Δ}\lambda_{\mathrm{outer}}}{\lambda }=1-\frac{n_{c}R}{n_{d}}\sqrt[{3}]{\frac{\left(1-\eta_{s}\right)}{R^{3}-\eta_{s}{\left(R-R_{\mathrm{res}}\right)}^{3}}}$$ (4)

Δλ_outer_ can be a negative value in the case that the cavity radius *R* is sufficiently large and the refractive index change dominates the responses.

As revealed in Fig. 2A, the blue shift trend ceases at the turning point *t* ~ 900 s, and a continuous red shift emerges. This observation indicates that *L* surpasses the radial extent of WGMs, and the sphere core is ingressed and gradually swelled [see Fig. 2B (right)]. The rate can be estimated by adopting Fickian’s law of diffusion

$$\frac{dL}{dt}=-K{\left(U-U_{\mathrm{th}}\right)}^{\gamma }$$ (5)

where the kinetic parameter *K* and the nonlinear coefficient γ are phenomenological constants, *U* is the penetrant molar concentration, and *U*_th_ is the concentration at a threshold level triggering the transition from the originally glassy state to the rubbery state. Because of the low concentration of penetrant molecules, the diffusion is very slow, and a saturation point of the red shift is not observed after ~4 hours of measurement.

**Fig. 3.**
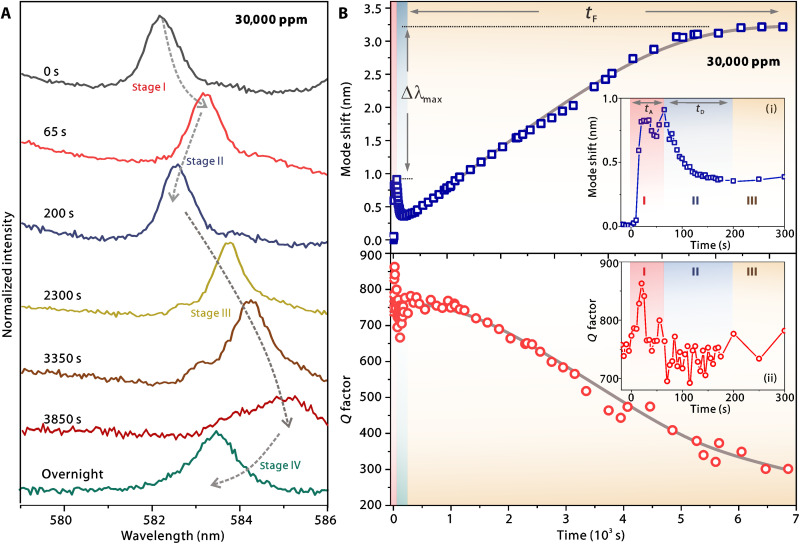
Evolution of resonance modes in a high-load condition. (**A**) Measured resonant spectra at different stages. (**B**) Mode wavelength shift (top) and *Q* factor (bottom) as a function of time upon a penetrant concentration of 30,000 ppm. Insets: Zoomed-in view of (B) at *t* = 0 to 300 s.

Further experiments were carried out at a largely increased ethanol concentration of 150-fold. One can find a clear-mode red shift at the beginning stage [*t* = 0 to 65 s; see Fig. 3 (A and B)], which is absent in the low-concentration case in Fig. 2A. By introducing a large number of penetrant molecules into the vicinity of the microsphere, physical absorption of molecules leads to a quick formation of a thin molecular film at the surface (*54*), and thus, the effective cavity radius is slightly increased up to an equilibrium reached at *t*_A_ ~ 65 s. In such a high-concentration case, the number of molecules captured per unit of time at the very initial stage (*t < t*_A_) is much larger than the diffused one, and hence, the accumulation of molecules on the surface leads to mode red shift.

For *t* = 65 to 200 s, the mode blue shift agrees with the observation in Fig. 2A, indicating that the diffusion at the outermost layer governs the response. One can note that the duration *t*_D_ (~115 s) is much shorter than that (~900 s) in the low gas loading condition (Fig. 2). This acceleration confirms that *dL*/*dt* is strongly dependent on the penetrant concentration. As implied in Eq. 5, the substantial increment of *U* speeds up the diffusion process by about eightfold. Despite the giant difference in concentration and *t*_D_ in the two cases, the measured Δλ_outer_ are still comparable (−0.36 nm in Fig. 2A and −0.55 nm in Fig. 3B). This is attributed to the similar solubility *U*_0_ at an unmodified temperature (considering that the laser heating effect is very minor here), and the characterized Δλ_outer_ values still follow the estimation with Eq. 4.

Similar to the accelerated diffusion at the outermost layer upon a high ethanol concentration, one can discern a relatively fast diffusion toward the sphere core. At *t* = 200 to 6900 s, the resonance is continuously red shifted until reaching a saturated value of Δλ_max_ = 2.3 nm (comparing to the wavelength at *t* = *t*_A_ while the initial equilibrium is established). While the polymer sphere is fully diffused (i.e., *L* = *R*), the maximum shift can be derived on the basis of Eq. 2 as follows

$$\frac{\text{Δ}\lambda_{\max}}{\lambda }=1-\frac{\sqrt[{3}]{n_{c}(1-\eta_{s})}}{n_{d}}$$ (6)

In addition to the tracking of resonant mode shift, the diffusion dynamics can also be understood by the evolution of *Q* factors. One can find a minor increment of *Q* from ~750 to ~870 at *t* = 0 to 20 s, which is consistent with previous reports studying molecular adsorption on a cavity surface (*54*). As the surface roughness could be reduced upon the formation of a uniform thin film, the scattering loss is potentially mitigated. A slight fluctuation of the *Q* factor around ~720 is observed at *t* = 65 to 200 s, followed by a notable degradation to ~300 at *t* = 200 to 6900 s. We attribute this to the spatial inhomogeneity of the diffusion and swelling process, as part of the microsphere close to (or in direct contact with) the substrate might not be well exposed to the environment. Hence, the originally circular-shaped cross section may get deformed, leading to increased optical scattering and leakage loss and an inhomogeneously broadened resonance lineshape (see detailed discussions in section S3). After the saturation point, the swelled microsphere is exposed to air overnight (~ 9 hours). The resonant wavelength is almost shifted back, and the high-*Q* resonance is also recovered. This indicates that penetrant molecules are released and the whole process is reversible, which agrees with the same process characterized using time-resolved reflection spectroscopy on distributed Bragg reflectors (*55*).

### Full process with retrieved parameters

**Fig. 4.**
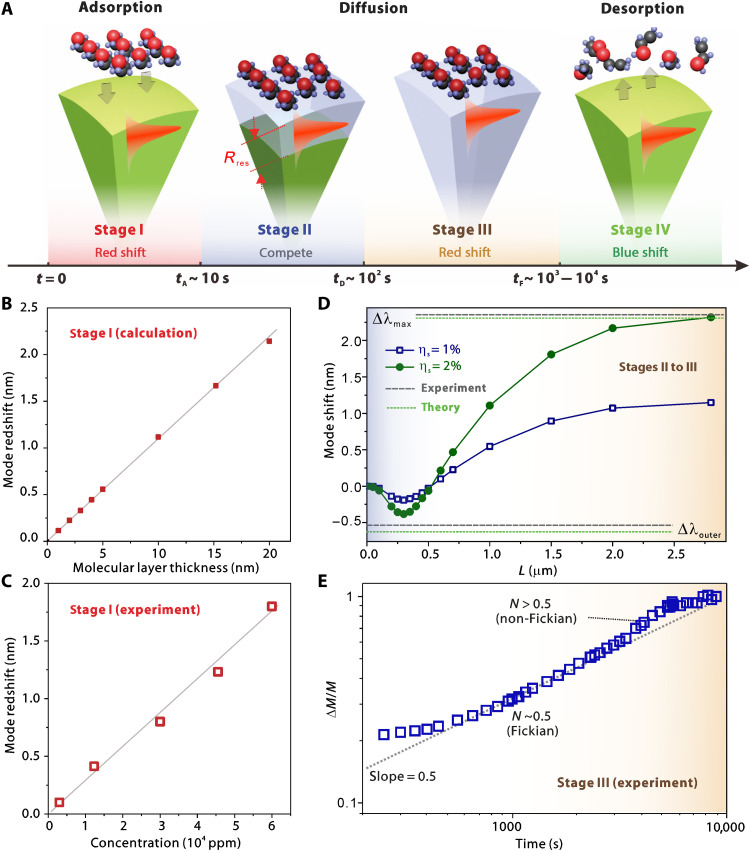
Overview of molecular diffusion dynamics with retrieved key parameters. (**A**) Summarized evolution of interactions between ethanol molecules and corresponding optical resonance signals, including adsorption, diffusion, and desorption. (**B**) Calculated mode shift as a function of molecular layer thickness using perturbation theory. (**C**) Measured mode red shift as a function of penetrant concentration. Dots: Experimental data. Line: A linear fit. (**D**) Numerically simulated mode shifts as a function of the depth of diffusion *L* upon η*_s_* of 1 and 2%. Two green dashed lines present the calculated values for η*_s_* = 2% using Eqs. 4 and 6. Two gray solid lines present the measured values in Fig. 3B. (**E**) Estimated mass uptake ratio as a function of time in a log-log scale.

Figure 4A shows an overview of the full process covering four stages. Stage I is the molecular adsorption at the polymer surface, occurring within the first few tens of seconds and leading to a quick spectral red shift. It is followed by stage II as a relatively long process of the initial diffusion at the outermost layer of the microsphere, in which the effects of Δ*n* and Δ*R* compete with each other. Stage III is the further inward diffusion up to an equilibrium with a fully swelling condition. Depending on the sphere size and also the molecular concentration, the duration *t*_F_ may take ~10- to 100-fold longer than *t*_D_ at stage II, leading to a continuous spectral red shift up to a saturated value and also a degradation of *Q* factor. At last, the penetrant molecules get desorbed on a long time scale, leading to the recovery of resonant wavelength and *Q* factor.

For surface adsorption at stage I, the mode red shift can be analyzed using perturbation theory

$$\frac{\text{Δ}\lambda }{\lambda }=\frac{\left\langle E(\vec{r})|\text{Δ}\varepsilon (\vec{r})|E(\vec{r})\right\rangle }{2\langle E(\vec{r})|\varepsilon (\vec{r})|E(\vec{r})\rangle }$$ (7)

where $E(\vec{r})$and $\varepsilon (\vec{r})$ are the distribution of electric field and permittivity, respectively. $\text{Δ}\varepsilon (\vec{r})$denotes the local variation of permittivity induced by the surface molecular layer. Here, two-dimensional (2D) numerical simulations based on the finite element method (COMSOL Multiphysics Wave Optics module) were used to calculate the mode response at stage I (see Materials and Methods). In Fig. 4B, the calculated mode shift is linearly proportional to the thickness of the thin molecular layer on the sphere surface. The estimated surface sensitivity is ~0.114 nm/nm.

To ascertain the origin of stage I, we repeat the studies upon different concentrations (see Fig. 4C). The red shift becomes spectrally resolved at sufficiently high concentrations (>1000 ppm), which explains the absence of initial red shift in Fig. 2. By summarizing the maximum mode shift at stage I as a function of the penetrant concentration, a nice linear relationship with a spectral sensitivity *S* of ~0.3 pm/ppm is obtained, which is consistent with the observations of surface sensing using both organic and inorganic WGM microcavities (*56*, *57*).

Here, numerical simulations were carried out to study the relationship between Δλ and *L* at stages II and III (see section S4 and fig. S10). Two sets of results with η*_s_* of 1 and 2% as examples are presented in Fig. 4D. For η*_s_* = 2%, the simulated Δλ_outer_ at *L* = 0.3 μm agrees nicely with the calculated value based on Eq. 4. Here, we would like to point out that the competition-governed mode response at stage II is dependent on multiple parameters, especially the exact mode field distribution. Here, the dependence on the optical polarization state is investigated by comparing the mode evolutions of both TM and TE modes (see section S5 and fig. S11). For the TM mode, the appearance of blue shift is a bit “delayed” compared to that of the TE mode, which agrees with the theory that the radial extent *R*_res_ for TM mode is slightly larger than that of the TE mode (fig. S11A). Besides, the competition at stage II is highly size dependent (see section S6). As implied by Eq. 4, the transition from red shift and blue shift occurs at *R* of ~2.2 μm (see fig. S12). With further experiments using microspheres with a reduced *R* ~ 1.5 μm, a distinct response at stage II is observed. Instead of mode blue shift, here, a moderate red shift is observed (fig. S13), which is further corroborated by the simulation results (fig. S14).

For stage III, the swelling-induced red shift gradually dominates the response and becomes saturated, while *L* approaches *R*. The simulated Δλ_max_ ~ 2.3 nm matches nicely with both the theoretical value based on Eq. 6. In practice, Δλ_max_ at equilibrium can be used to extract the actual volume fraction (or solubility) of ethanol in PS. According to Flory-Huggins theory, while the polymer matrix is fully swollen, the thermodynamic equilibrium condition can be written as (*45*)

$$\ln\eta_{s}+(1\text{-}\eta_{s})+\chi {\left(1\text{-}\eta_{s}\right)}^{2}=0$$ (8)

where χ is the dimensionless Flory-Huggins interaction parameter between polymer and penetrant. χ varies linearly with 1*/T_e_* and thus can be expressed as χ = *a + b/T_e_*, where *a* and *b* are empirical parameters. According to previous gravimetric measurements (*45*), the volume fraction of ethanol in PS at 20°C is calculated as 2.3%, which agrees nicely with our estimated value (~2%) probed by optical resonances.

For stage III in which the swelling solely governs the mode shift, the diffusion kinetics can be further understood by assessing the time course of diffused mass. Considering the relative mass uptake $\frac{\text{Δ}M(t)}{\text{Δ}M_{\max}}$ in a polymer-penetrant system (*58*), a power-law expression can be written as

$$\frac{\text{Δ}M(t)}{\text{Δ}M_{\max}}∼\frac{\text{Δ}R(t)}{\text{Δ}R_{\max}}=\frac{\text{Δ}\lambda (t)-\lambda \text{Δ}n/n}{\text{Δ}\lambda_{\max}-\lambda \text{Δ}n/n}={Ct}^{N}$$ (9)

where *C* is a constant and *N* determines the diffusion behavior, namely, Fickian diffusion (*N* = 0.5), non-Fickian diffusion (0.5 < *N* < 1), and case II diffusion (*N* = 1).

As summarized in a log-log plot in Fig. 4E, for Δ*M*/Δ*M*_max_ < 0.5, a smooth *t*^0.5^ dependence is observed (*58*). One can estimate *D_g_* at a glassy PS through fitting with the following equation

$$\frac{\text{Δ}M(t)}{\text{Δ}M_{\max}}∼\frac{\text{Δ}\lambda (t)-\lambda \text{Δ}n/n}{\text{Δ}\lambda_{\max}-\lambda \text{Δ}n/n}=\frac{2}{R}\sqrt{\frac{D_{g}t}{\pi }}$$ (10)

The extracted diffusion coefficient *D_g_* here is ~10^−11^ cm^2^/s, showing a good agreement with previous reports using flat polymer films and gravimetric methods (*11*, *12*). As a slow effect at *T_e_* << *T_g_*, *D_g_* is about two orders lower than the values above *T_g_*. Notably, instead of a saturation followed by a plateaus region, a slope > 0.5 is observed, indicating the non-Fickian contribution of the sorption. Such a phenomenon with dynamics beyond the Fickian equilibration has also been observed by the conventional gravimetric method long ago (*49*).

## DISCUSSION

In summary, we propose and experimentally demonstrate a route of studying diffusion dynamics in polymer microspheres using optical resonances. While conventional WGM microcavity sensors focus on detecting specific or nonspecific adsorptions onto the surface, our report shows that optical resonances can be pivoted to understanding microscopic thermodynamics. A complete picture containing four stages of interaction in a penetrant-microsphere system is unveiled and quantitatively depicted by tracking the spectral shift and linewidth change of resonances. Assuming a thermodynamic equilibrium can be gradually reached, the solubility of penetrant in polymer at a particular temperature can be retrieved by virtue of the tracked mode evolution. Compared with other previously developed optical techniques assessing diffusion dynamics, here our WGM resonance-enabled approach offers noninvasive, in situ precise measurements for understanding diffusion dynamics with clearly revealed time courses, which is particularly advantageous for miniaturized systems of glassy polymer far below *T_g_* with potentially low solubility of solvent.

The technique can be further applied to studying microsystems interacting with various types of organic or inorganic molecules in either ambient or aqueous environments. Moreover, our demonstrated in situ route could be versatile and applicable to a big variety of polymer microstructures (e.g., polygons, cylinders, disks, and tubes) supporting WGM resonances, which avoids the complexity of designing and fabricating distributed Bragg reflectors in previously reported Flory-Huggins sensors (*11*, *59*, *60*). The polymer structure is not limited to miniaturized objects with a size of a few micrometers. Polymer-based WGM cavities with a larger size (up to about millimeters) and those with alternative designs such as freestanding ones or polymer-coated cavities (*61*, *62*), could be particularly favorable in leveraging ultrahigh-*Q* resonances (*Q* ~ 10^4^ to 10^9^) and resolving tiny changes at different stages throughout the interaction. All in all, the work is envisaged to unveil diffusion dynamics in a noninvasive manner by resonant light probing and also unleash vast potential applications to tap in, for instance, the drug load/release performance in delivery systems, the degradation of environmental pollutants, and the lifetime assessment of polymer devices.

## MATERIALS AND METHODS

### Sample preparation

The mature chemical synthesis technology of monodisperse microspheres offers good homogeneity, high-quality surface morphology, and also flexibility in surface functionalization. In experiments, commercially available monodisperse PS microspheres doped with rhodamine B dyes (1% w/v; So-Fe Biomedicine) were adopted. The microsphere surface was functionalized with a carboxyl (COOH) group. The characterized glass transition temperature is ~94°C. Two sets of PS microsphere samples with a diameter of 6 and 3 μm (coefficient of variance, <10%) were studied. The 1:100 diluted suspension of microspheres was drop-cast onto a quartz substrate and sealed into a homemade chamber with a volume of ~0.7 cm^3^. The chamber was constructed with a substrate, a spacer, and a 0.17-mm-thick cover glass and sealed by ultraviolet-curing optical adhesives (NOA 68). Microfluidic PTFE Teflon tubing was integrated into the inlet and outlet of the chamber. Gas samples containing a mixture of ethanol and air with different concentrations were prepared in 1-liter Teflon bags and delivered to the chamber by a syringe. The ethanol concentration varies from 200 ppm to the highest saturable value (~60,000 ppm).

### Optical characterizations

In situ spectral measurements were conducted using a confocal microscopic PL system. Excitation was conducted using a continuous-wave (CW) laser (Cobolt Samba, 532 nm) and a long-working distance objective lens (Olympus LMPLFLN 50×; numerical aperture, 0.5) with a spot size ≈ 1 μm^2^. For long-time measurements of molecular interactions, resonant modes in PL signals upon CW pumping were analyzed instead of lasing modes (fig. S3) upon pulsed pumping due to their better stability. The focusing spot was aligned to the rim of individual microspheres to maximize the excitation and collection efficiency of WGMs. The power of laser excitation onto the sample was adjusted to ~0.4 μW using a neutral density filter (optical density, 4) to avoid any photobleaching and potential heating effect, which may perturb the WGMs and cause additional resonance drifts. The emission light was guided to the spectrometer with 600 blz/mm and an electrically cooled charge-coupled device camera. The polarization state was determined using a rotatable half-wave plate and a fixed polarization analyzer. Spatially resolved mapping was performed using a motorized stage with a step of 0.2 μm.

### Numerical simulation

2D numerical simulations were performed on the basis of the finite-element method (COMSOL Multiphysics Wave Optics module). A circular-shaped perfect matching layer as the outermost boundary was introduced for simulating the mode field distribution. Fine meshing with a size of ~2 nm was applied to the outermost region of the cavity overlapping with the resonant optical fields. The eigenmodes and eigenfrequencies for both TM and TE modes around 590 to 600 nm were numerically solved and tracked upon varying conditions of sorption and diffusion.

## Supplementary Materials

### This PDF file includes:

Sections S1 to S6 Figs. S1 to S14 References
