# Supplementary material for: Revealing molecular diffusion dynamics in polymer microspheres by optical resonances
Source: Sci Adv. 2023 May 10;9(19):eadf1725. doi: 10.1126/sciadv.adf1725 (PMC10171802; doi:10.1126/sciadv.adf1725)
Supplement: Supplementary file 1 — Sections S1 to S6 Figs. S1 to S14 References [file sciadv.adf1725_sm.pdf]

Science Advances
AAAS

Supplementary Materials for

Revealing molecular diffusion dynamics in polymer microspheres by optical resonances

Jiawei Wang *et al.*

Corresponding author: Jiawei Wang, wangjw7@hit.edu.cn; Engui Zhao, zhaoengui@hit.edu.cn;
Yong Sheng Zhao, yszhao@iccas.ac.cn

Sci. Adv. **9**, eadf1725 (2023)
DOI: 10.1126/sciadv.adf1725

This PDF file includes:

Sections S1 to S6
Figs. S1 to S14
References

Section 1. The Bruggeman model for estimating the refractive index

The Bruggeman model is usually adopted to analyze a composite system containing two or more materials that are randomly interspersed (*52*). Assuming that the grain of each material is spherical. The internal electric field E_i upon a uniformly applied field E_0 can be estimated as follow:

$$E_{\mathrm{i}} = \frac{3\varepsilon_{\mathrm{eff}}}{\varepsilon_{\mathrm{i}} + 2\varepsilon_{\mathrm{eff}}} E_0 . \tag{S1}$$

where ε_i is the purely real dielectric permittivity of constituents and ε_{eff} is the effective dielectric constant that falls between the minimum and maximum values of ε_i for each material. Hence, the displacement field can be written as:

$$D_{\mathrm{i}} = \varepsilon_{\mathrm{i}} \frac{3\varepsilon_{\mathrm{eff}}}{\varepsilon_{\mathrm{i}} + 2\varepsilon_{\mathrm{eff}}} E_0 . \tag{S2}$$

Assuming the system contains only two materials (the solvent and host in our case), the average displacement field of the composite can be estimated by taking the weighted sum of the individual fields,

$$D = \varepsilon_{\mathrm{eff}} E_0 = \eta_1 D_1 + \eta_2 D_2 , \tag{S3}$$

where η_1 and η_2 are the volume fraction of two materials, respectively. Considering the volume fraction of core polymer (η_c) and solvent molecule (η_s), the following relation can be derived:

$$0 = \eta_s \frac{\varepsilon_s - \varepsilon_{\mathrm{eff}}}{\varepsilon_s + 2\varepsilon_{\mathrm{eff}}} + \eta_c \frac{\varepsilon_c - \varepsilon_{\mathrm{eff}}}{\varepsilon_c + 2\varepsilon_{\mathrm{eff}}} . \tag{S4}$$

The permittivity of the linear effective system can be derived:

$$\varepsilon_{\mathrm{eff}} = [(4\varepsilon_c{}^2\eta_c{}^2 - 4\varepsilon_c{}^2\eta_c\eta_s + \varepsilon_c{}^2\eta_s{}^2 + 4\varepsilon_c\varepsilon_s\eta_c{}^2 + 26\varepsilon_c\varepsilon_s\eta_c\eta_s + 4\varepsilon_c\varepsilon_s\eta_s{}^2 + \varepsilon_s{}^2\eta_c{}^2 - 4\varepsilon_s{}^2\eta_c\eta_s + 4\varepsilon_s{}^2\eta_s{}^2)^{(1/2)} + 2\varepsilon_c\eta_c - \varepsilon_c\eta_s - \varepsilon_s\eta_c + 2\varepsilon_s\eta_s] / [4(\eta_c + \eta_s)] \tag{S5}$$

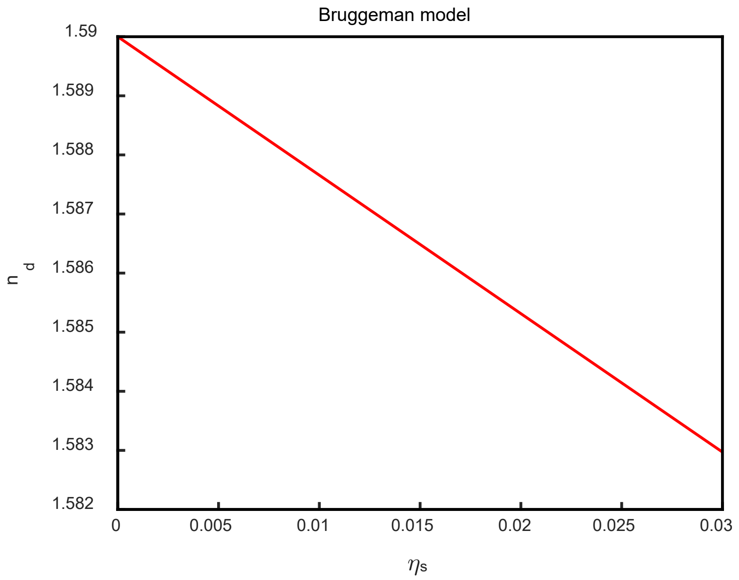

Fig. S1. Calculated relationship between η_s and refractive index n_d.

The refractive index of the diffused layer can be expressed as $n_d = \sqrt{\varepsilon_{\mathrm{eff}}}$. Figure S1 shows the calculated n_d as a function of η_s. For this polymer-penetrant system, the refractive index change can be estimated as follow:

$$\frac{\Delta n}{n} = \frac{n_d - n_c}{n_c}, \tag{S6}$$

where n_c is the refractive index of the undiffused core layer.

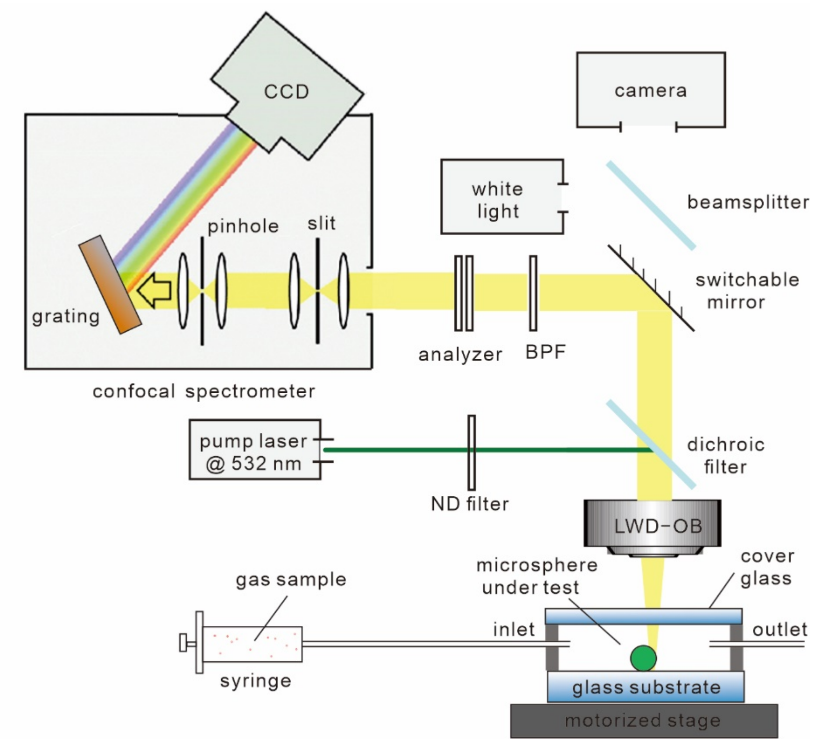

Fig. S2. Schematic of the experimental setup. BPF: bandpass filter. LWD-OB: long working distance objective lens. ND filter: neutral-density filter.

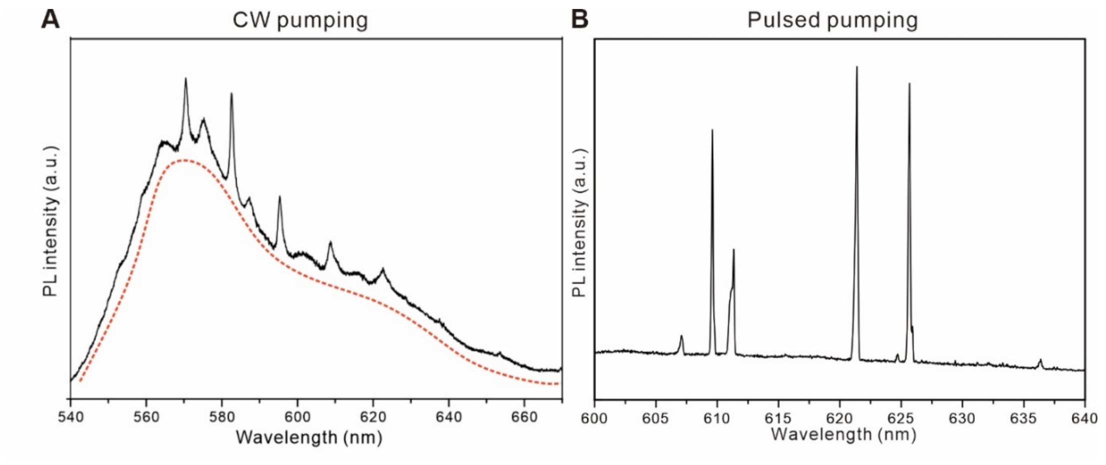

Fig. S3. Resonant spectra under CW and pulsed pumping. (**A**) Measured PL spectrum under CW pumping. The red dashed curve denotes the spontaneous emission as background signals. (**B**) Measured lasing spectrum under pulsed pumping.

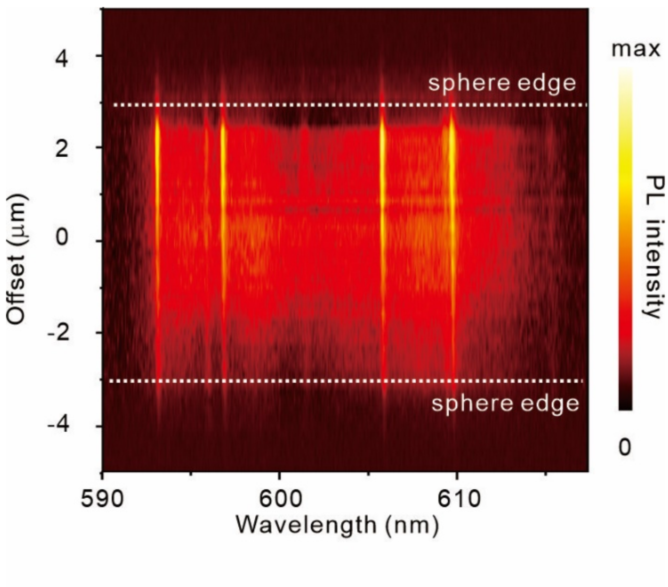

Fig. S4. **Spatially and spectrally resolved image showing resonances.** TM- and TE- polarized resonance modes of a 6 μm-sized PS microsphere can be discerned.

Section 2. Theory of mode response considering both swelling and relaxation effects of polymer

While the solvent starts to diffuse into an originally dry polymer, the phase transition may occur along with the process of polymer relaxation (*42*). For tracking of optical resonances, both swelling and relaxation may contribute to the changes in the sphere size and refractive index. Here the theory of mode response considering both effects is presented, while the simplified theory presented in the main article ignores the effect of polymer relaxation.

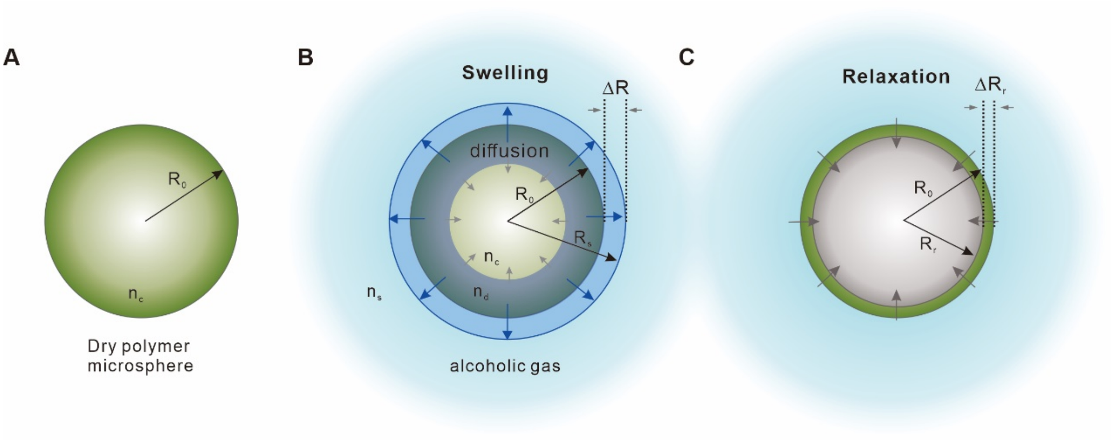

Fig. S5. Schematic showing a microsphere before and after interacting with ethanol molecules. (**A**) a dry polymer microsphere, (**B**) a microsphere interacting with ethanol showing the swelling effect, and (**C**) a microsphere experiencing polymer relaxation.

As illustrated in Fig. S5A-B, the expanded volume of the sphere due to swelling can be estimated accordingly. The radius R_s at the saturation equilibrium can be written as:

$$R_s = R_0 / \left(\sqrt[3]{1-\eta_s} \right), \tag{S7}$$

where R_0 is the sphere radius in the unswollen state, and η_s is the volume fraction of solvent in the polymer at equilibrium. Hence, the radius change can be expressed as:

$$\Delta R = R_s - R_0 = \left(\frac{1}{(1-\eta_s)^{\frac{1}{3}}} - 1 \right) R_0 . \tag{S8}$$

Given the constraint of optical resonances $n\omega R \approx$ const, the response of resonant wavelength can be given:

$$\frac{\Delta\lambda}{\lambda} = 1 - \frac{1}{\left(1+\frac{\Delta n}{n}\right)\left(1+\frac{\Delta R}{R}\right)} \approx \frac{\Delta n}{n} + \frac{\Delta R}{R} . \tag{S9}$$

Combining with Eqs. S8 and S9, the maximum resonance shift at the end of stage III (i.e., saturation equilibrium) can be derived as:

$$\frac{\Delta\lambda_{\max}}{\lambda} = 1 - \frac{\sqrt[3]{n_c (1-\eta_s)}}{n_d} . \tag{S10}$$

One should note that the actual size of the diffused polymer sphere may not exactly match the volume sum of the polymer and penetrant. This is attributed to the relaxation of the polymer itself (*63*). The slow relaxation leads to a continuous change in the volume and also the associated refractive index. For amorphous polymers, the volume can be calculated from the atomic radius and bond length of the constituent atoms following the approach proposed by Slonimskii et al (*64*). Packing coefficient K is defined for estimating the molecular volume of a specific amorphous polymer. The actual molecule volume of a monomer unit V is expressed as:

$$V = \frac{V_{\text{int}}}{K} , \tag{S11}$$

where V_{int} is the intrinsic molecular volume considering only the total volume of individual atoms.

The refractive index of the polymer-based compound can be calculated based on the Lorentz-Lorenz equation,

$$\text{n} = \sqrt{\left(2\frac{[R]}{V}+1\right) / \left(1-\frac{[R]}{V}\right)} , \tag{S12}$$

where [R] is the molar refraction (*65*). Besides, The refractive index can be readily characterized using prism-coupling measurement (*63*). Previous studies suggest the measured n in the range of ~ 1.588 to ~1.589 during the enthalpy relaxation of PS at varying temperatures (*66*). Based on Eqs. S11 and S12, the dependence of the refractive index on the packing coefficient can be calculated in Fig. S6, revealing a clear linear relationship with dn/dK of 1.129 RIU (*66*).

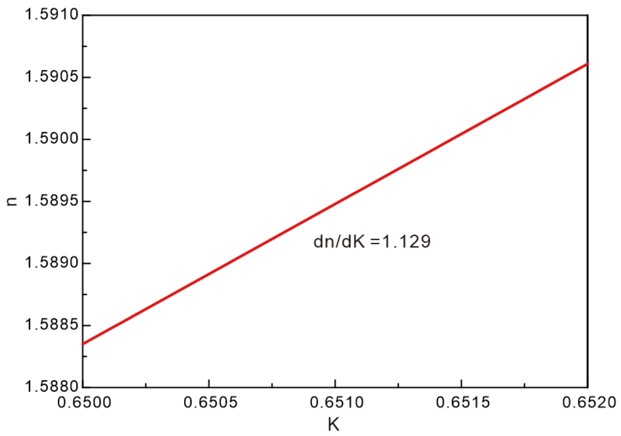

Fig. S6. The calculated dependence of refractive index *n* on packing coefficient *K*.

Assuming a maximum change of K is reached upon relaxation at the final thermodynamic equilibrium state, the radius R_r due to relaxation can be written as:

$$R_{\mathrm{r}} = \left(\sqrt[3]{1 + \frac{\Delta K}{K_0}} \right)^{-1} R_0 , \tag{S13}$$

where K_0 is the original packing coefficient.

Here, the overall refractive index change considering both swelling and relaxation effects can be expressed as:

$$\frac{\Delta n}{n} = \frac{n_d - n_c + \frac{dn}{dK} \Delta K}{n_c} . \tag{S14}$$

Combining with Eqs. S13 and S14, the maximum resonance shift can be derived as:

$$\frac{\Delta\lambda_{\max}}{\lambda}=1-\frac{n_c R\left[\left(1+\frac{\Delta K}{K_0}\right)^{-\frac{1}{3}}+\left(1-\eta_s\right)^{-\frac{1}{3}}-1\right]^{-1}}{n_d+\frac{dn}{dK}\Delta K}. \tag{S15}$$

In the following, the scenario of stage II in which the diffusion is limited at the outermost layer is discussed. Under this circumstance, the swelling effect is limited at the mode volume of the optical resonance with the depth $L = R_{\text{res}}$.

$$R_s^{\,3}-R_0^3=\eta_s\left[R_s^{\,3}-\left(R_0-R_{\text{res}}\right)^3\right]. \tag{S16}$$

The radius change can be expressed as follows:

$$\Delta R\ =R_{\text{s}}-R_0=\sqrt[3]{\frac{R_0^{\,3}-\eta_s\left(R_0-R_{\text{res}}\right)^3}{1-\eta_s}}-R_0. \tag{S17}$$

According to Eqs. S14 and S17, while only the diffusion and swelling process is considered, the maximum resonant shift at stage II can be derived as:

$$\frac{\Delta\lambda_{\text{outer}}}{\lambda}=1-\frac{n_c R}{n_d}\sqrt[3]{\frac{\left(1-\eta_s\right)}{R^3-\eta_s\left(R-R_{\text{res}}\right)^3}}\ . \tag{S18}$$

Now the relaxation process is taken into account. Similar to polymer swelling, the polymer relaxation only occurs at the outermost layer, while the inner core layer remains to be glassy. The overall sphere volume V can be expressed as the sum of the unperturbed core V_{core} and perturbed outermost layer V_{res}:

$$V=V_{\text{core}}+V_{\text{res}}+\Delta V_{\text{res}}=V_{\text{core}}+\frac{V_{\text{res}}}{1+\frac{\Delta K}{K_0}}. \tag{S19}$$

Hence, the relaxation-induced radius change ΔR_r can be readily derived,

$$\Delta R_{\text{r}}=\sqrt[3]{\left(R_0-R_{\text{res}}\right)^3+\frac{R_0^3-\left(R_0-R_{\text{res}}\right)^3}{1+\frac{\Delta K}{K_0}}}-R_0 \tag{S20}$$

Based on Eq. S14 and Eq. S20, the maximum resonant shift at stage II can be derived as:

$$\frac{\Delta\lambda_{\text{outer}}}{\lambda}=1-\frac{n_cR}{n_d+\frac{dn}{dK}\Delta K}\left[\sqrt[3]{\left(R_0-R_{\text{res}}\right)^3+\frac{R_0^3-\left(R_0-R_{\text{res}}\right)^3}{1+\frac{\Delta K}{K_0}}}+\sqrt[3]{\frac{R_0{}^3-\eta_s\left(R_0-R_{\text{res}}\right)^3}{1-\eta_s}}-R_0\right]^{-1} \quad \text{(S21)}$$

Here theoretical calculations are made based on empirical values for both relaxation and swelling effects. The volume relaxation conditions for PS and other common polymers have been carefully characterized in previous studies at varied temperature (*63*). Considering the most exaggerated case of maximum volume relaxation of PS in previous experimental studies using different methods (e.g., dilatometer) (*53, 66*), ΔK reaches ~0.001. Here we define some of the basic parameters according to the experimental configurations, namely n_c=1.59, λ=590 nm, R=3 μm, and R_{res}=0.3 μm, and also empirical values of K_0 (0.65) from a previous report (*66*). Therefore, the resonance shifts can be theoretically studied as a function of ΔK and η_s. Figure S7A-B summarizes the theoretically calculated $\Delta\lambda_{outer}$ and $\Delta\lambda_{max}$ upon a reasonable variation of ΔK (0 – 0.001) and η_s (0 – 0.03) of interactions between PS and ethanol according to literature (*53, 66*).

Here the theory is applied to understanding the experimental results in Fig. 3A. Considering the most exaggerated case of ΔK ~0.001 (*53*), Fig. S7C-D reveals the extracted η_s of ~ 2.2 % ± 0.53 % and ~ 2.01% ± 0.05 % upon measured values of $\Delta\lambda_{outer}$ (-0.55 nm) and $\Delta\lambda_{max}$ (2.3 nm), respectively. One can conclude that the polymer relaxation plays a minor role in the optical responses, especially $\Delta\lambda_{max}$ at the saturation equilibrium. Therefore, a simplified theory neglecting the effect of polymer relaxation is presented in the main article focusing on the extraction of η_s.

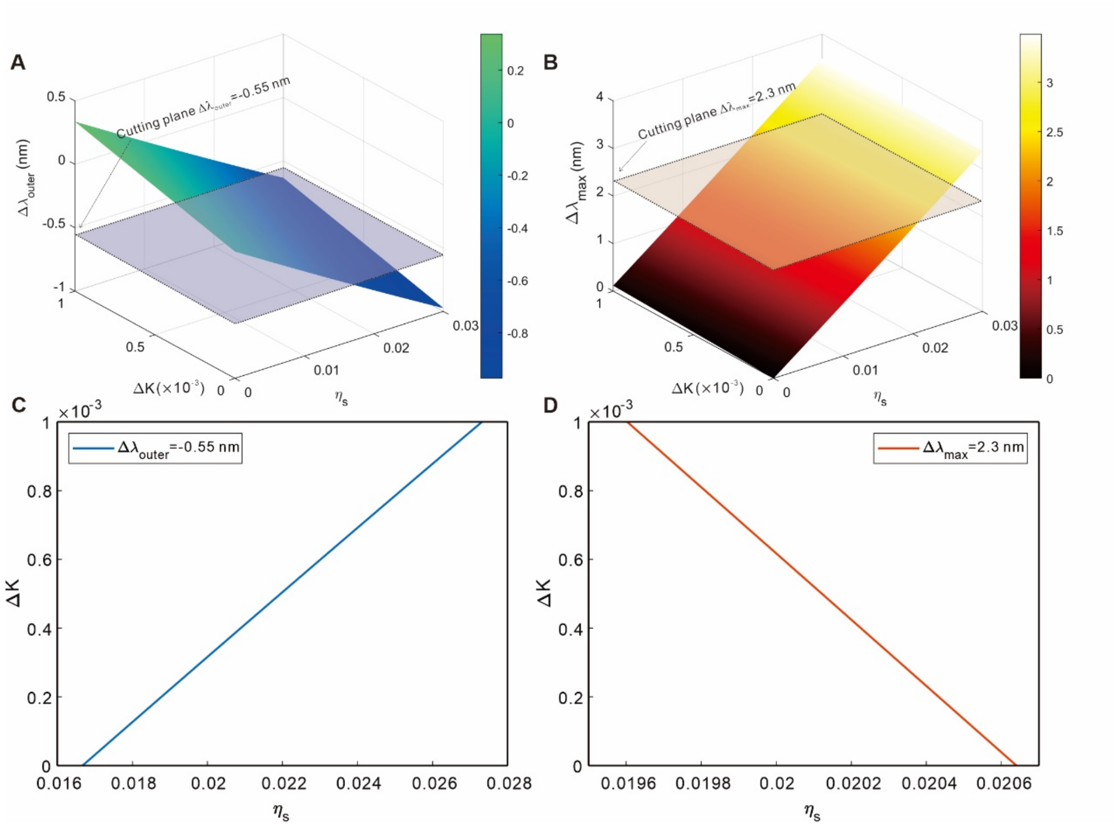

Fig. S7. Calculated $\Delta\lambda_{outer}$ and $\Delta\lambda_{max}$ as a function of ΔK and η_s. (**A**) Calculated $\Delta\lambda_{outer}$ as a function of ΔK and η_s. (**B**) Calculated $\Delta\lambda_{max}$ as a function of ΔK and η_s. (**C**) Cutting plane at $\Delta\lambda_{outer}$ of -0.55 nm in (**A**). (**B**) Cutting plane at $\Delta\lambda_{max}$ of 2.3 nm in (**B**).

Section 3. Substrate-induced effects on optical resonances and mode responses

In practice, studying molecular diffusion dynamics using freestanding polymer microspheres is technically non-trivial. Here substrate-induced effects on optical resonances and mode responses to diffusion are discussed. For microspheres as a 3D WGM microcavity system, the resonant orbits are not restricted to a fixed plane. The left panel of Fig. S8A illustrates two orthogonal paths along which the light goes around the circumference in the horizontal and vertical planes, respectively. Without a substrate, the modes with the same polarization and resonating along distinct orbits are spectrally degenerate. For microspheres placed on a substrate, the resonance in the vertical plane gets substantially perturbed. In contrast, the substrate leads to negligible perturbation to the resonance in the horizontal plane, which has been carefully examined in previous spatially-resolved spectroscopies (*67*).

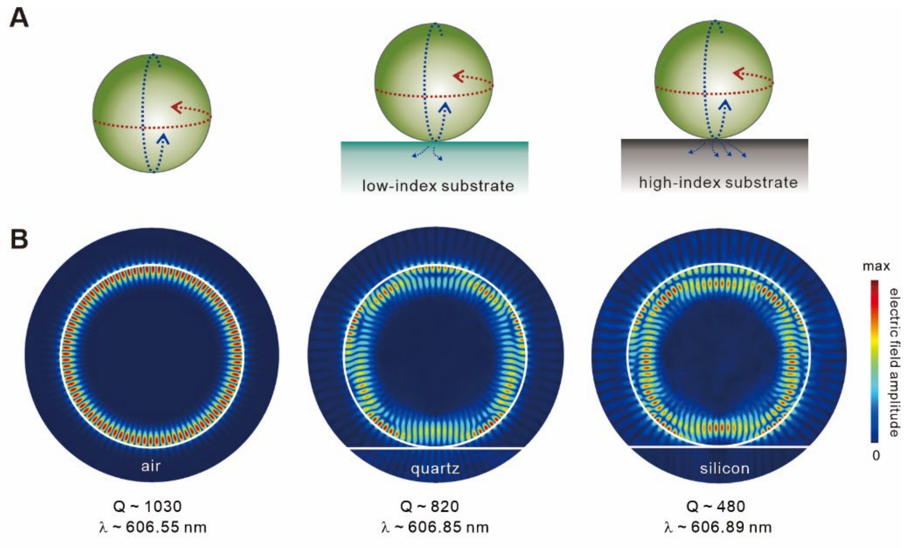

Fig. S8. Schematics and simulated field distributions of microspheres without and with a substrate. (**A**) Schematics showing two resonant orbits with orthogonal paths in a freestanding microsphere (left) and a microsphere placed on a low-index (middle) or a high-index (right) substrate. (**B**) Simulated TE-polarized mode field amplitude distributions: without a substrate (left), with a quartz (n = 1.46, middle) and a silicon (n = 3.97, right) substrate.

The substrate-induced perturbation can be readily examined using numerical simulations. Figure S8B summarizes the simulated TE-polarized mode profile without and with a substrate as an example. Compared to the case without a substrate (left panel), the modes in the vertical plane become spectrally redshifted, and also much lossy due to substrate leakage. The mode profile of preserved resonances reveals a clear difference from a regular WGM with the lowest (fundamental) radial mode order. The Q factor further decreases by changing a transparent, low-index substrate to a high-index substrate with strong absorption in the visible wavelength (see the middle and right panel).

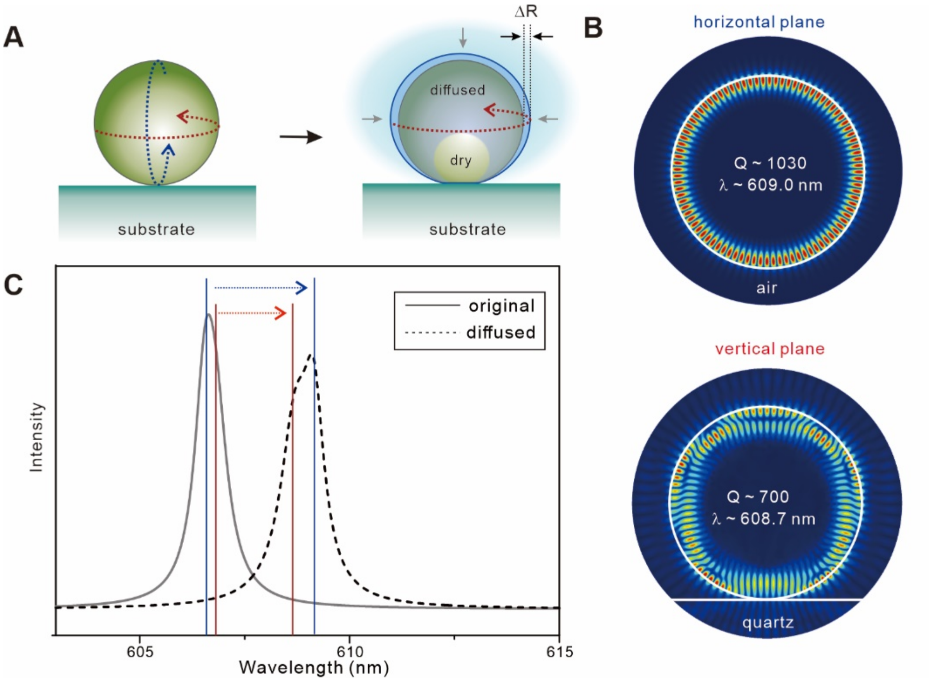

Fig. S9. Simulated mode responses considering an inhomogeneous diffusion. (**A**) Schematic showing the spatial inhomogeneity of the diffusion and swelling process due to the existence of substrate. (**B**) Simulated TE-polarized mode field amplitude distribution along the horizontal plane (fully diffused) and vertical plane (partially diffused) upon η_s = 2 %. (**C**) Simulated resonant spectra show the spectral shift and linewidth change due to the inhomogeneous broadening effect. The blue and red lines denote the modes with resonant orbits along the horizontal and vertical planes, respectively.

In experiments studying polymer-solvent interactions, part of the microsphere close to (or in direct contact with) the substrate is not fully exposed to the environment. This

leads to spatial inhomogeneity of the diffusion and swelling process, as illustrated in Fig. S9A. Here numerical simulation is carried out upon the scenario that part of the sphere close to the substrate is not fully diffused (Fig. S9B). For the mode resonating in the horizontal plane, the isotropic swelling leads to $\Delta\lambda_{max} \sim 2.4$ nm, which agrees with the observation in Fig. 4B in the main article. In contrast, regarding the mode resonating in the vertical plane, the originally circular-shaped cross-section gets slightly deformed. This results in $\Delta\lambda_{max} \sim 1.9$ nm and a degradation of the Q factor due to the ascending scattering loss and substrate leakage.

Figure S9C presents the simulated resonant spectra considering both modes resonating at two planes before and after the diffusion. The overall resonance lineshape gets broadened due to the broken spectral degeneracy of two resonances. The decrease in the Q factor is attributed to the inhomogeneous broadening effect of perturbed resonances at two planes, which agrees with the observation in Fig. 3B. Nevertheless, one can discern that the spectral shift is dominated by the long-lived mode at the horizontal plane in which the effect of the substrate can be neglected. Therefore, the technique is applicable to studies of diffusion in polymer microspheres with the existence of a substrate.

Section S4. Numerical simulation of mode response upon varying *L*

The resonances of a microsphere with R = 3 μm upon diffusion of ethanol molecules with a varying depth of L are calculated (the effect of substrate is not considered here), so as to extract the mode shift in Fig. 4D in the main article. Regular TE-polarized WGM at azimuthal mode order M = 44 is discerned in the mode field profile in Figure S10. While L reaches R_{res}, the mode shift turns from a blueshift to a redshift. Here the radial extent $L = R_{res} \sim 0.3$ μm is highly consistent with the calculated value based on Eq. 3 in the main article.

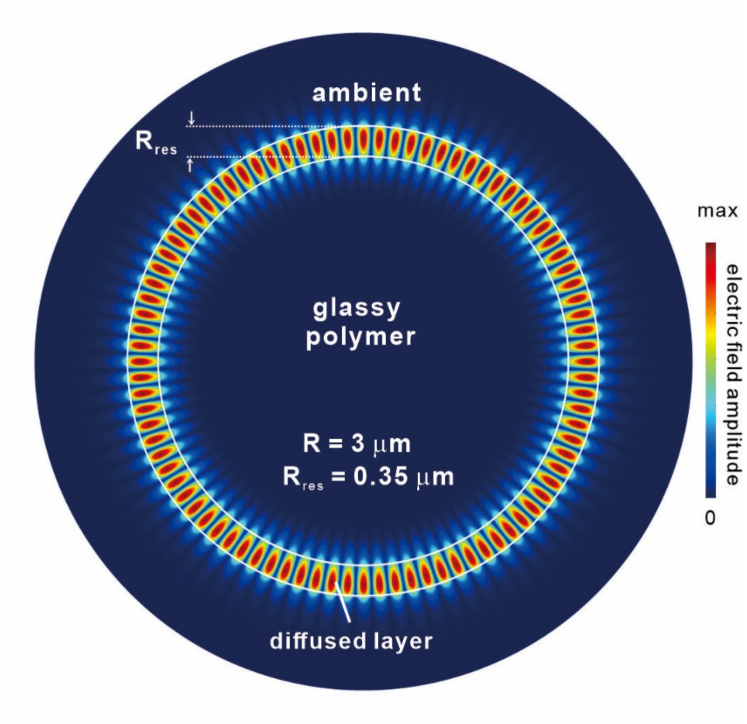

Fig. S10. Simulated electric field amplitude distribution at the turning point of the spectral shift. The turning point between stages II and III is reached upon L = R_{res}. The two concentric white circles denote R_{res}.

Section S5. Polarization-dependent mode responses

It is well understood that TM and TE polarized WGMs feature distinct mode field distributions along the radial direction (see Fig. S11A), which lead to polarization-dependent responses in such a penetrant-microsphere system. As to stage I, the stronger evanescent field of TM mode leads to a slightly higher surface sensitivity and a larger redshift rather than those of TE mode. As to stage II, one can discern a time delay for TM mode to "feel" the expansion and perturbation of the refractive index compared with that of TE mode (Fig. S11B). As revealed in the simulated profile in Fig. S11A, the overall TM mode field is slightly inner towards the core than that of TE mode, which alters the competition of ΔR and Δn. Simulated results upon varying L in Fig. S11C suggest that $\Delta\lambda_{outer}$ at stage II in TM mode is less compared to that in TE mode.

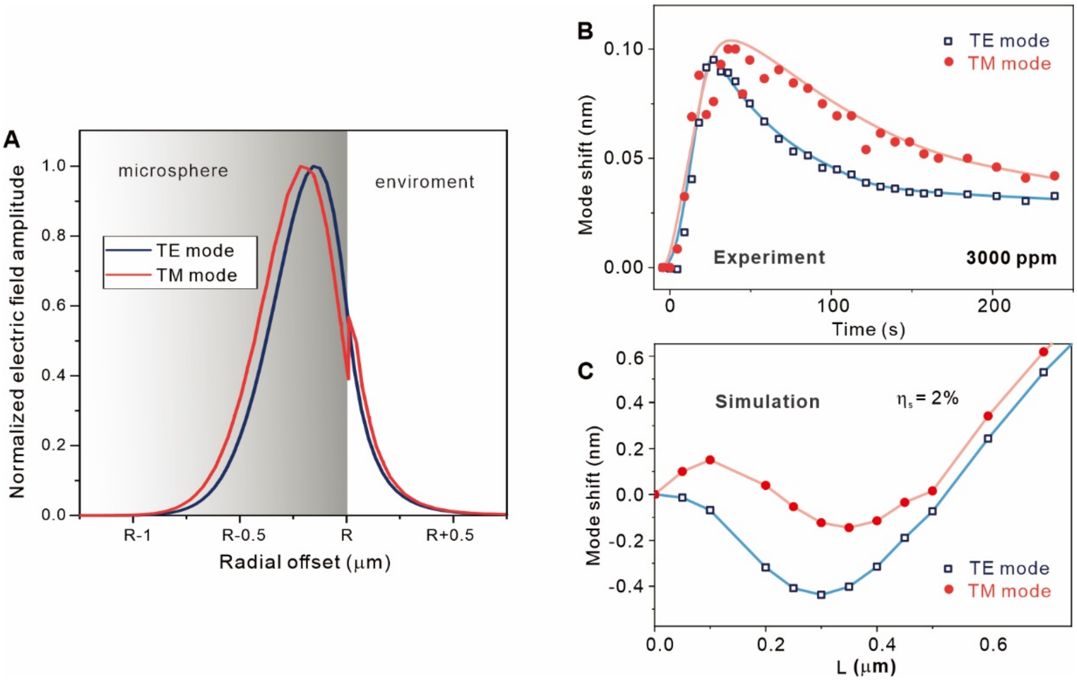

Fig. S11. Simulated and measured mode evolution upon two polarizations. (**A**) Numerically simulated mode field amplitude along the radial direction. (**B**) The extracted mode wavelength shift for both TM and TE modes as a function of time upon a concentration of 3000 ppm. (**C**) Calculated mode shifts as a function of L for both TM and TE modes upon $\eta_s = 2$ %.

Section S6. Size-dependent mode responses

For stage II, the dependence of $\Delta\lambda_{outer}$ on the sphere size is studied based on Eqs. 3 and 4 in the main article. As summarized in Fig. S12, a smaller R leads to a reinforced effect of ΔR, and hence a redshift ($\Delta\lambda_{outer} > 0$). For $R > 2.2$ mm, the effect of Δn gradually governs the response and contributes to a blueshift ($\Delta\lambda_{outer} < 0$).

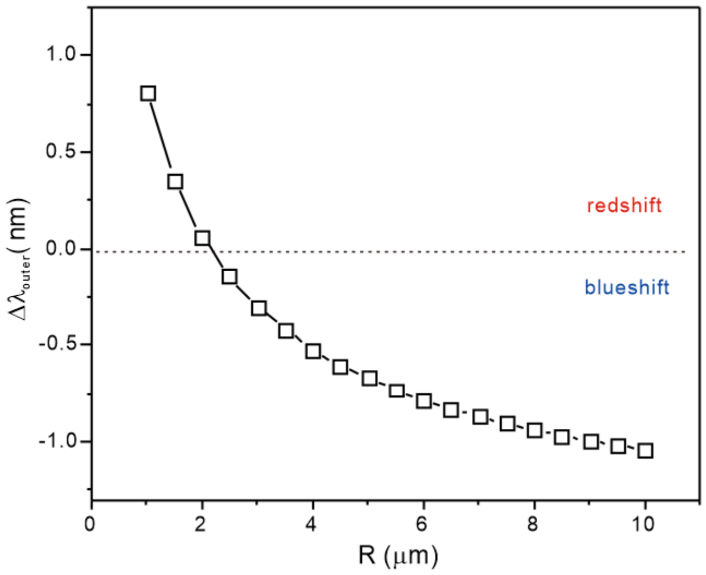

Fig. S12. **Calculated mode shift at stage II as a function of cavity radius *R*.**

The dependence was verified by both numerical simulation and experiments. Fig. S13A presents the TE-polarized WGM field profile ($M = 20$) of a microsphere with $R = 1.5$ μm. In contrast with Fig. 3B, one can discern a very moderate redshift at the initial stage of diffusion ($L < R_{res}$) in Fig. S13B. The extracted $\Delta\lambda_{outer}$ at $L = R_{res} \sim 0.5$ nm agrees nicely with the theoretical value predicted by Eq. 4.

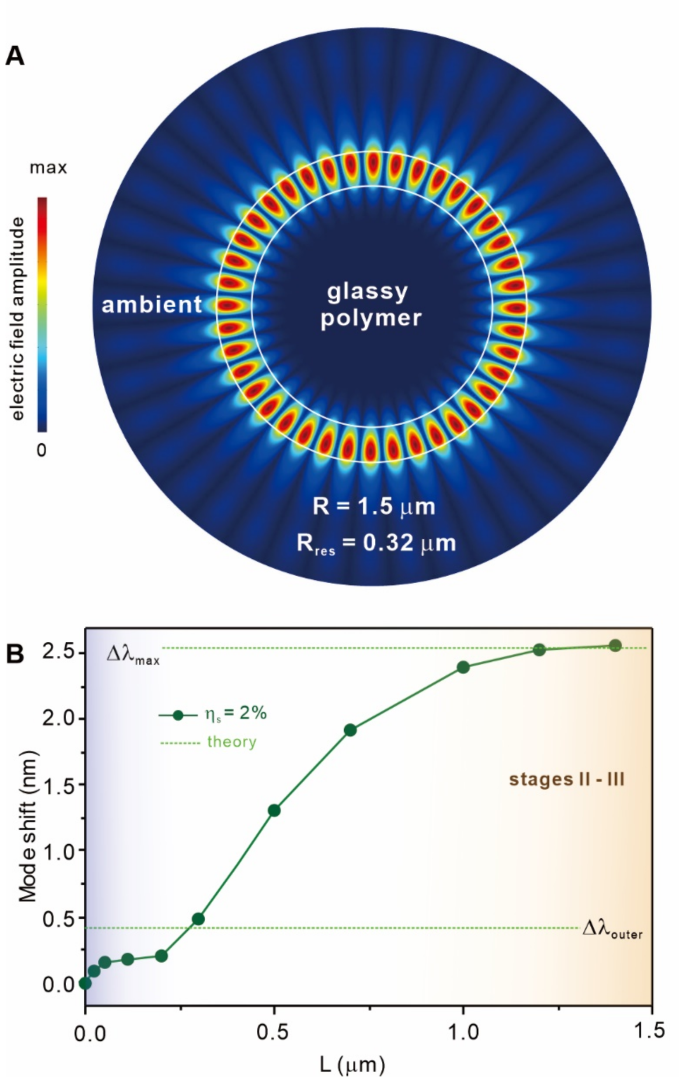

Fig. S13. Simulated mode distribution and mode shift for a microsphere with *R* = 1.5 µm. (**A**) Simulated electric field amplitude distribution of a microsphere (R = 1.5 µm) while a turning point between stage II and III is reached. The two concentric white circles denote R_{res}. (**B**) Numerically simulated mode shifts as a function of L. Two green dashed lines present the calculated values using Eqs. 4 and 7 in the main article.

Besides, the extracted $\Delta\lambda_{max}$ in both simulations and theoretical calculations in Fig. S13B match with the one of R = 3 µm (see Fig. 4D in the main article). This again indicates that the saturated value at stage III is solely dependent on η_s, which offers a straightforward way to measure the solubility based on characterizing $\Delta\lambda_{max}$.

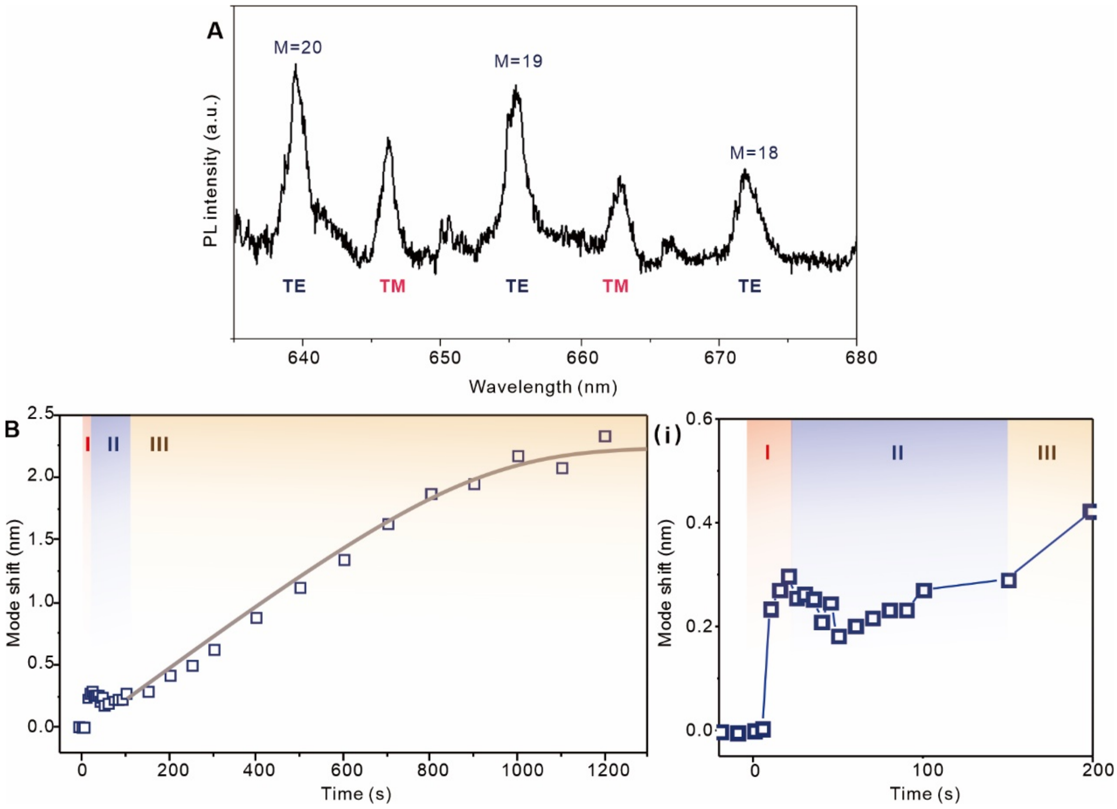

Fig. S14. **Measured mode evolution for a microsphere with *R* = 1.5 μm.** (**A**) Measured resonant mode spectrum of a microsphere (*R* = 1.5 μm) containing both TM and TE modes with different azimuthal mode orders. (**B**) Mode wavelength shift as a function of time upon a concentration of 8000 ppm. Inset: zoomed-in view at *t* = 0 - 200 s.

For experiments, the same measurements were repeated on another set of microspheres with *R* reduced from 3 to 1.5 μm (see Fig. S14A). Figure S14B shows the evolution of mode shift covering stages I - III. After the initial redshift of ~ 0.25 nm at stage I, Δλ fluctuates between 0.2 - 0.3 nm as a consequence of the competing effect, which examines the simulated effect in Fig. S13B. After *t* = 150 s, the redshift is rapidly built up, which reveals the inward diffusion at the core region at stage III.

REFERENCES AND NOTES

1. N. Kamaly, B. Yameen, J. Wu, O. C. Farokhzad, Degradable controlled-release polymers and polymeric nanoparticles: Mechanisms of controlling drug release. *Chem. Rev.* **116**, 2602–2663 (2016).

2. Y. Shi, L. Peng, Y. Ding, Y. Zhao, G. Yu, Nanostructured conductive polymers for advanced energy storage. *Chem. Soc. Rev.* **44**, 6684–6696 (2015).

3. F. A. A. Nugroho, I. Darmadi, L. Cusinato, A. Susarrey-Arce, H. Schreuders, L. J. Bannenberg, A. B. da Silva Fanta, S. Kadkhodazadeh, J. B. Wagner, T. J. Antosiewicz, A. Hellman, V. P. Zhdanov, B. Dam, C. Langhammer, Metal–polymer hybrid nanomaterials for plasmonic ultrafast hydrogen detection. *Nat. Mater.* **18**, 489–495 (2019).

4. T. P. Gall, R. C. Lasky, E. J. Kramer, Case II diffusion: Effect of solvent molecule size. *Polymer* **31**, 1491–1499 (1990).

5. D. Pierleoni, M. Minelli, G. Scherillo, G. Mensitieri, V. Loianno, F. Bonavolonta, F. Doghieri, Analysis of a polystyrene-toluene system through "dynamic" sorption tests: Glass transitions and retrograde vitrification. *J. Phys. Chem. B* **121**, 9969–9981 (2017).

6. G. Mensitieri, G. Scherillo, C. Panayiotou, P. Musto, Towards a predictive thermodynamic description of sorption processes in polymers: The synergy between theoretical EoS models and vibrational spectroscopy. *Mater. Sci. Eng. R Rep.* **140**, 100525 (2020).

7. D. Wang, C. He, M. P. Stoykovich, D. K. Schwartz, Nanoscale topography Influences polymer surface diffusion. *ACS Nano* **9**, 1656–1664 (2015).

8. J. E. Saunders, H. Chen, C. Brauer, M. Clayton, W. Chen, J. A. Barnes, H.-P. Loock, Quantitative diffusion and swelling kinetic measurements using large-angle interferometric refractometry. *Soft Matter* **11**, 8746–8757 (2015).

9. T.-M. Chang, S. S. Xantheas, A. E. Vasdekis, Mesoscale polymer dissolution probed by Raman spectroscopy and molecular simulations. *J. Phys. Chem. B* **120**, 10581–10587 (2016).

10. Y. Zhang, Q. Fu, J. Ge, Photonic sensing of organic solvents through geometric study of dynamic reflection spectrum. *Nat. Commun.* **6**, 7510 (2015).

11. P. Lova, G. Manfredi, C. Bastianini, C. Mennucci, F. Buatier de Mongeot, A. Servida, D. Comoretto, Flory–Huggins photonic sensors for the optical assessment of molecular diffusion coefficients in polymers. *ACS Appl. Mater. Interfaces* **11**, 16872–16880 (2019).

12. P. Lova, H. Megahd, D. Comoretto, Thin polymer films: Simple optical determination of molecular diffusion coefficients. *ACS Appl. Polym. Mater.* **2**, 563–568 (2019).

13. Y. A. Elabd, M. G. Baschetti, T. A. Barbari, Time-resolved Fourier transform infrared/attenuated total reflection spectroscopy for the measurement of molecular diffusion in polymers. *J. Polym. Sci. B Polym. Phys.* **41**, 2794–2807 (2003).

14. F. Vollmer, D. Braun, A. Libchaber, M. Khoshsima, I. Teraoka, S. Arnold, Protein detection by optical shift of a resonant microcavity. *Appl. Phys. Lett.* **80**, 4057–4059 (2002).

15. X. Jiang, A. J. Qavi, S. H. Huang, L. Yang, Whispering-gallery sensors. *Matter* **3**, 371–392 (2020).

16. D. Yu, M. Humar, K. Meserve, R. C. Bailey, S. N. Chormaic, F. Vollmer, Whispering-gallery-mode sensors for biological and physical sensing. *Nat. Rev. Methods Primers* **1**, 83 (2021).

17. X.-C. Yu, S.-J. Tang, W. Liu, Y. Xu, Q. Gong, Y.-L. Chen, Y.-F. Xiao, Single-molecule optofluidic microsensor with interface whispering gallery modes. *Proc. Natl. Acad. Sci. U.S.A.* **119**, e2108678119 (2022).

18. Y. Zhang, C. Zhang, Y. Fan, Z. Liu, F. Hu, Y. S. Zhao, Smart protein-based biolasers: An alternative way to protein conformation detection. *ACS Appl. Mater. Interfaces* **13**, 19187–19192 (2021).

19. T. Reynolds, N. Riesen, A. Meldrum, X. Fan, J. M. M. Hall, T. M. Monro, A. François, Fluorescent and lasing whispering gallery mode microresonators for sensing applications. *Laser Photonics Rev.* **11**, 1600265 (2017).

20. J. Wang, D. Karnaushenko, M. Medina-Sánchez, Y. Yin, L. Ma, O. G. Schmidt, Three-dimensional microtubular devices for lab-on-a-chip sensing applications. *ACS Sens.* **4**, 1476–1496 (2019).

21. J. Wang, Y. Yin, Q. Hao, Y. Zhang, L. Ma, O. G. Schmidt, Strong coupling in a photonic molecule formed by trapping a microsphere in a microtube cavity. *Adv. Opt. Mater.* **6**, 1700842 (2018).

22. K. Scholten, X. Fan, E. T. Zellers, A microfabricated optofluidic ring resonator for sensitive, high-speed detection of volatile organic compounds. *Lab Chip* **14**, 3873–3880 (2014).

23. K. J. Vahala, Optical microcavities. *Nature* **424**, 839–846 (2003).

24. F. Vollmer, S. Arnold, Whispering-gallery-mode biosensing: Label-free detection down to single molecules. *Nat. Methods* **5**, 591–596 (2008).

25. S. Subramanian, H.-Y. Wu, T. Constant, J. Xavier, F. Vollmer, Label-free optical single-molecule micro- and nanosensors. *Adv. Mater.* **30**, e1801246 (2018).

26. S. Zhang, S.-J. Tang, S. Feng, Y.-F. Xiao, W. Cui, X. Wang, W. Sun, J. Ye, P. Han, X. Zhang, Y. Zhang, High-Q polymer microcavities integrated on a multicore fiber facet for vapor sensing. *Adv. Opt. Mater.* **7**, 1900602 (2019).

27. Y. Kong, Z. Zhao, Y. Wang, S. Yang, G. Huang, Y. Wang, C. Liu, C. You, J. Tan, C. Wang, B. Xu, J. Cui, X. Liu, Y. Mei, Integration of a metal-organic framework film with a tubular whispering-gallery-mode microcavity for effective CO_2 sensing. *ACS Appl. Mater. Interfaces*, **48**, 58104–58113 (2021).

28. M. Gao, C. Wei, X. Lin, Y. Liu, F. Hu, Y. S. Zhao, Controlled assembly of organic whispering-gallery-mode microlasers as highly sensitive chemical vapor sensors. *Chem. Commun.* **53**, 3102–3105 (2017).

29. A. R. Ali, C. M. Elias, Ultra-sensitive optical resonator for organic solvents detection based on whispering gallery modes. *Chemosensors* **5**, 2227–9040 (2017).

30. C. Lemieux-Leduc, R. Guertin, M.-A. Bianki, Y.-A. Peter, All-polymer whispering gallery mode resonators for gas sensing. *Opt. Express* **29**, 8685–8697 (2021).

31. W. Y. Heah, H. Yamagishi, K. Fujita, M. Sumitani, Y. Mikami, H. Yoshioka, Y. Oki, Y. Yamamoto, Silk fibroin microspheres as optical resonators for wide-range humidity sensing and biodegradable lasers. *Mater. Chem. Front.* **5**, 5653–5657 (2021).

32. A. Qiagedeer, H. Yamagishi, M. Sakamoto, H. Hasebe, F. Ishiwari, T. Fukushima, Y. Yamamoto, A highly sensitive humidity sensor based on an aggregation-induced emission luminogen-appended hygroscopic polymer microresonator. *Mater. Chem. Front.* **5**, 799–803 (2021).

33. A. Qiagedeer, H. Yamagishi, S. Hayashi, Y. Yamamoto, Polymer optical microcavity sensor for volatile organic compounds with distinct selectivity toward aromatic hydrocarbons. *ACS Omega* **6**, 21066–21070 (2021).

34. S. Avino, A. Krause, R. Zullo, A. Giorgini, P. Malara, P. De Natale, H. P. Loock, G. Gagliardi, Direct sensing in liquids using whispering-gallery-mode droplet resonators. *Adv. Opt. Mater.* **2**, 1155–1159 (2014).

35. L. Burratti, F. De Matteis, M. Casalboni, R. Francini, R. Pizzoferrato, P. Prosposito, Polystyrene photonic crystals as optical sensors for volatile organic compounds. *Mater. Chem. Phys.* **212**, 274–281 (2018).

36. C. Fenzl, T. Hirsch, O. S. Wolfbeis, Photonic crystals for chemical sensing and biosensing. *Angew. Chem. Int. Ed. Engl.* **53**, 3318–3335 (2014).

37. T. Endo, Y. Yanagida, T. Hatsuzawa, Colorimetric detection of volatile organic compounds using a colloidal crystal-based chemical sensor for environmental applications. *Sens. Actuators B Chem.* **125**, 589–595 (2007).

38. W. Zhang, J. Yao, Y. S. Zhao, Organic micro/nanoscale lasers. *Acc. Chem. Res.* **49**, 1691–1700 (2016).

39. J. Wang, Q. Hao, Y. Yin, L. Ma, O. G. Schmidt, Surface-enhanced Raman scattering enabled by metal-coated dielectric microspheres. *Phys. Status Solidi B Basic Res.* **256**, 1800379 (2019).

40. T. Wienhold, S. Kraemmer, S. F. Wondimu, T. Siegle, U. Bog, U. Weinzierl, S. Schmidt, H. Becker, H. Kalt, T. Mappes, S. Koeber, C. Koos, All-polymer photonic sensing platform based on whispering-gallery mode microgoblet lasers. *Lab Chip* **15**, 3800–3806 (2015).

41. D.-Q. Yang, J.-H. Chen, Q.-T. Cao, B. Duan, H.-J. Chen, X.-C. Yu, Y.-F. Xiao, Operando monitoring transition dynamics of responsive polymer using optofluidic microcavities. *Light. Sci. Appl.* **10**, 128 (2021).

42. M. R. Foreman, F. Vollmer, Optical tracking of anomalous diffusion kinetics in polymer microspheres. *Phys. Rev. Lett.* **114**, 118001 (2015).

43. S. Schiller, Asymptotic expansion of morphological resonance frequencies in Mie scattering. *Appl. Optics* **32**, 2181–2185 (1993).

44. O. J. Karlsson, J. M. Stubbs, L. E. Karlsson, D. C. Sundberg, Estimating diffusion coefficients for small molecules in polymers and polymer solutions. *Polymer* **42**, 4915–4923 (2001).

45. G. Bernardo, D. Vesely, Equilibrium solubility of alcohols in polystyrene attained by controlled diffusion. *Eur. Polym. J.* **43**, 938–948 (2007).

46. N. E. Schlotter, Diffusion of small molecules in glassy polymer thin films studied by waveguide Raman techniques. *J. Phys. Chem.* **94**, 1692–1699 (1990).

47. T. Alfrey Jr., E. F. Gurnee, W. G. Lloyd, Diffusion in glassy polymers. *J. Polym. Sci. Polym. Symp.* **12**, 249–261 (1966).

48. G. Scherillo, M. Galizia, P. Musto, G. Mensitieri, Water sorption thermodynamics in glassy and rubbery polymers: Modeling the interactional issues emerging from FTIR spectroscopy. *Ind. Eng. Chem. Res.* **52**, 8674–8691 (2012).

49. A. R. Berens, H. B. Hopfenberg, Diffusion of organic vapors at low concentrations in glassy PVC, polystyrene, and PMMA. *J. Membr. Sci.* **10**, 283–303 (1982).

50. M. S. Eroğlu, O. Güven, Characterization of network structure of poly(glycidyl azide) elastomers by swelling, solubility and mechanical measurements. *Polymer* **39**, 1173–1176 (1998).

51. G. B. McKenna, J. A. Hinkley, Mechanical and swelling behaviour of well characterized polybutadiene networks. *Polymer* **27**, 1368–1376 (1986).

52. R. J. Gehr, R. W. Boyd, Optical properties of nanostructured optical materials. *Chem. Mater.* **8**, 1807–1819 (1996).

53. K. Adachi, T. Kotaka, Volume and enthalpy relaxation in polystyrene. *Polym. J.* **14**, 959–970 (1982).

54. Y. Yin, J. Wang, X. Wang, S. Li, M. R. Jorgensen, J. Ren, S. Meng, L. Ma, O. G. Schmidt, Water nanostructure formation on oxide probed in situ by optical resonances. *Sci. Adv.* **5**, eaax6973 (2019).

55. S. Gao, X. Tang, S. Langner, A. Osvet, C. Harreiss, M. K. S. Barr, E. Spiecker, J. Bachmann, C. J. Brabec, K. Forberich, Time-resolved analysis of dielectric mirrors for vapor sensing. *ACS Appl. Mater. Interfaces* **10**, 36398–36406 (2018).

56. L. Ma, S. Li, V. A. Quinones, L. Yang, W. Xi, M. Jorgensen, S. Baunack, Y. Mei, S. Kiravittaya, O. G. Schmidt, Dynamic molecular processes detected by microtubular opto-

chemical sensors self-assembled from prestrained nanomembranes. *Adv. Mater.* **25**, 2357–2361 (2013).

57. A. K. Mallik, G. Farrell, D. Liu, V. Kavungal, Q. Wu, Y. Semenova, Silica gel coated spherical micro resonator for ultra-high sensitivity detection of ammonia gas concentration in air. *Sci. Rep.* **8**, 1620 (2018).

58. G. Bernardo, Diffusivity of alcohols in amorphous polystyrene. *J. Appl. Polym. Sci.* **127**, 1803–1811 (2013).

59. H. Megahd, P. Lova, D. Comoretto, Universal design rules for Flory–Huggins polymer photonic vapor sensors. *Adv. Funct. Mater.* **31**, 2009626 (2021).

60. H. Megahd, C. Oldani, S. Radice, A. Lanfranchi, M. Patrini, P. Lova, D. Comoretto, Aquivion–poly(*N*-vinylcarbazole) holistic Flory–Huggins photonic vapor sensors. *Adv. Opt. Mater.* **9**, 2002006 (2021).

61. K. Gardner, Y. Zhi, L. Tan, S. Lane, Y. F. Xiao, A. Meldrum, Whispering gallery mode structure in polymer-coated lasing microspheres. *J. Opt. Soc. Am. B* **34**, 2140–2146 (2017).

62. D. Yang, A. Wang, J.-H. Chen, X.-C. Yu, C. Lan, Y. Ji, Y.-F. Xiao, Real-time monitoring of hydrogel phase transition in an ultrahigh *Q* microbubble resonator. *Photon. Res.* **8**, 497–502 (2020).

63. N. Tanio, T. Nakanishi, Physical aging and refractive index of poly(methyl methacrylate) glass. *Polym. J.* **38**, 814–818 (2006).

64. A. A. Askadskii, G. L. Slonimskii, Universal system of calculation for determining the glass transition temperature of polymers. *Polym. Sci. U.S.S.R.* **13**, 2158–2160 (1971).

65. H. Looyenga, Dielectric constants of homogeneous mixture. *Mol. Phys.* **9**, 501–511 (1965).

66. N. Tanio, T. Nakanishi, Physical aging and refractive index of optical polymer glass. *Kobunshi Ronbunshu* **66**, 31–35 (2009).

67. T. Mitsui, Y. Wakayama, T. Onodera, Y. Takaya, H. Oikawa, Observation of light propagation across a 90 corner in chains of microspheres on a patterned substrate. *Opt. Lett.* **33**, 1189–1191 (2008).
